# The interaction of two novel putative proteins of *Leptospira interrogans* with E-cadherin, plasminogen and complement components with potential role in bacterial infection

**DOI:** 10.1080/21505594.2019.1650613

**Published:** 2019-08-19

**Authors:** Leandro T. Kochi, Luis G. V. Fernandes, Gisele O. Souza, Silvio A. Vasconcellos, Marcos B. Heinemann, Eliete C. Romero, Karin Kirchgatter, Ana L. T. O. Nascimento

**Affiliations:** a Laboratório Especial de Desenvolvimento de Vacinas, Instituto Butantan, São Paulo, Brazil; b Programa de Pós-Graduação Interunidades em Biotecnologia, Instituto de Ciências Biomédicas, São Paulo, Brazil; c Laboratório de Zoonoses Bacterianas, Faculdade de Medicina Veterinária e Zootecnia, São Paulo, Brazil; d Centro de Bacteriologia, Instituto Adolfo Lutz, Sao Paulo, Brazil; e Núcleo de Estudos em Malária, Superintendência de Controle de Endemias -SUCEN/IMT-SP, USP, Sao Paulo, Brazil

**Keywords:** Leptospira, leptospirosis, recombinant proteins, adhesion, immune evasion

## Abstract

Leptospirosis is a worldwide zoonosis caused by pathogenic species of *Leptospira*. Leptospires are able to adhere to exposed extracellular matrix in injured tissues and, once in the bloodstream, can survive the attack of the immune system and spread to colonize target organs. In this work, we report that two novel putative proteins, coded by the genes LIC11711 and LIC12587 of *L. interrogans* serovar Copenhageni are conserved among pathogenic strains, and probably exposed in the bacterial surface. Soluble recombinant proteins were expressed in *Escherichia coli*, purified and characterized. Both recombinant proteins bound to laminin and E-cadherin, suggesting an initial adhesion function in host epithelial cells. The recombinant protein LIC11711 (rLIC11711) was able to capture plasminogen (PLG) from normal human serum and convert to enzymatically active plasmin (PLA), in the presence of PLG activator. rLIC12587 (recombinant protein LIC12587) displayed a dose dependent and saturable interaction with components C7, C8, and C9 of the complement system, reducing the bactericidal effect of the complement. Binding to C9 may have consequences such as C9 polymerization inhibition, interfering with the membrane attack complex formation. Blocking LIC11711 and LIC12587 on bacterial cells by the respective antiserum reduced leptospiral cell viability when exposed to normal human serum (NHS). Both recombinant proteins could be recognized by serum samples of confirmed leptospirosis, but not of unrelated diseases, suggesting that the native proteins are immunogenic and expressed during leptospirosis. Taken together, our data suggest that these proteins may have a role in leptospiral pathogenesis, participating in immune evasion strategies.

## Introduction

Leptospirosis is a widespread zoonosis of global importance whose etiological agents are pathogenic bacteria of the genus *Leptospira*. Rodents play an important role in the life cycle of leptospirosis, acting as main reservoirs of the disease, since they are chronic carriers, harboring leptospires in their kidneys, excreting them alive in the environment and contaminating water and soil [1]. In humans, the bacteria penetrate mainly through cuts and abrasions on the skin and mucous membranes when exposed to the contaminated medium [2]. Symptoms of the disease can be mild, like headaches, fever and muscle aches, even more severe such as jaundice and kidney failure that characterize Weil’s syndrome [3] and hemorrhagic pulmonary syndrome [4].

Despite all efforts, there is still no effective vaccine against leptospirosis. Available veterinary vaccines are used in livestock and in companion animals, while in a few countries human vaccination is restricted to groups that work in areas of risk [5–8]. Elucidating the functional role of various proteins that are expressed during the infectious process may help to understand the pathogenesis of leptospires.

It is known that the process of initial leptospiral adhesion to biomolecules present in the cells that make up the epithelial tissue is a fundamental step for the success of the bacterial host invasion and colonization. In this aspect, the interaction with several extracellular matrix components (ECM) and to surface cell-receptors has already been investigated for leptospires by our group [9–14]. Another mechanism employed by pathogenic leptospires is the binding to circulating host plasma components. Plasminogen (PLG) is a highly studied zymogen present in plasma and characterized as a ligand of leptospiral surface proteins. PLG can be converted into its active form, plasmin (PLA), a broad-spectrum serine protease capable of degrading immune mediators and ECM, allowing bacterial immune evasion and tissue invasion for dissemination through the bloodstream [15]. Furthermore, leptospiral proteins interact with regulators or components of the complement system pathways present in host serum as a possible mechanism to evade the innate immune system and lytic activity [16,17]. Interaction of *Leptospira* to fibrinogen (Fg) leading to a partial inhibition of fibrin clotting formation through Fg/thrombin-catalyzed reaction may help the bacterial dissemination [18]. Several proteins have the capacity to binding Fg but only in a few cases this interaction leads to fibrin clot inhibition [14,19].

In this work, we selected two hypothetical lipoproteins of unknown functions, previously detected and quantified in whole leptospiral cell extract by mass spectrometry [20], encoded by LIC11711 and LIC12587 of *L. interrogans* serovar Copenhageni. The genes were cloned and the recombinant proteins, expressed in *Escherichia coli*, characterized. Our results indicate that both proteins are surface-exposed, capable of interacting with several host components, suggesting that they could have importance in the pathogenesis of leptospirosis.

## Materials and methods

### Bacterial strains and serum samples

The pathogenic virulent strain *L. interrogans* serovar Copenhageni strain Fiocruz L1-130, pathogenic culture-attenuated of *L. interrogans* serovar Copenhageni strain M20, *L. interrogans* serovar Canicola strain Hound Utrecht IV; *L. interrogans* serovar Icterohaemorrhagiae strain RGA; *L. interrogans* serovar Pomona strain Pomona; *L. borgpetersenii* serovar Whitticombi strain Whitticombi; *L. kirschneri* serovar Cynopteri strain 3522C; *L. kirschneri* serovar Grippotyphosa strain Moskva V; *L. santarosai* serovar Shermani strain 1342 K and the non-pathogenic *L. biflexa* serovar Patoc strain Patoc1 were employed. In order to avoid unnecessary risks, the virulent strain was used only when necessary since our data showed that the proteins of this study are expressed in culture-attenuated *Leptospira*. Bacteria were cultured at 28°C under aerobic conditions in liquid EMJH medium (Difco, Franklin Lakes, USA) [21] modified with rabbit serum (10%; v/v). *E. coli* DH5α and *E. coli* BL21 (DE3) Star pLysS (Invitrogen, Carlsbad, USA) were used as cloning and recombinant protein expression hosts, respectively. Serum samples of patients diagnosed with leptospirosis (at both onset and convalescent phase) or other febrile diseases (dengue, malaria, Chagas disease and HIV) were from the serum collection of Instituto Adolfo Lutz, São Paulo, Brazil, and Núcleo de Estudos em Malária, Superintendência de Controle de Endemias -SUCEN/IMT-SP, USP, Brazil. These samples were donated for research purposes only.

### Extracellular matrix and plasma components

Laminin (L2020), collagen types I and IV (C3867 and C0543), cellular fibronectin (F2518), plasma fibronectin (F2006), Fg (F4883), PLG (P7999), elastin (E6902), E-cadherin (5085), thrombin (T6884), vitronectin (V8379) and bovine serum albumin (BSA; A3912) were acquired from Sigma-Aldrich. Laminin and collagen type IV were derived from the basement membrane of Engelbreth-Holm-Swarm mouse sarcoma; collagen type I was isolated from rat-tail; PLG, Fg, thrombin, and vitronectin were purified from human plasma; fibronectin was derived from human foreskin fibroblasts; elastin was derived from human aorta. Factor H was purified from human serum (341274, EMD Chemicals). C4b, C4BP, C6, C7, C8, and C9 were isolated from normal human serum (Complement Technology).

### In silico *sequences analysis*

The LIC11711 (LIC_RS08730) and LIC12587 (LIC_RS13250) coding sequences (CDSs) – accession numbers AAS70300.1 and AAS71148.1, respectively, were identified in the genome sequences of *L. interrogans* serovar Copenhageni [22,23] and selected based on the prediction of their cellular location by the software CELLO [24] and PSORT [25]; LipoP [26] was used to predict the presence of signal peptide. Sequence similarity was performed using BLASTp webserver [27].

### Cloning, expression, and purification of recombinant LIC11711 and LIC12587

The genes were amplified without the putative signal peptide sequence by PCR using the genomic DNA of *L. interrogans* serovar Copenhageni M20 strain as template and specific oligonucleotides (Table 1). The amplicons were purified and cloned into the pGEM-T Easy (Promega Corporation) and subcloned into the pAE vector [28] at the restriction sites BamHI and KpnI. All cloned sequences were confirmed by automated sequencing. Expression of the recombinant proteins rLIC11711 and rLIC12587 was performed in *E. coli* BL21 (DE3) Star pLysS with 1mM Isopropyl β-D-1-thiogalactopyranoside (IPTG, 420322, Calbiochem). Recombinant proteins were purified from soluble fraction of *E. coli* lysates by metal chelating chromatography, as previously described [29]. Fractions from all chromatographic steps were analyzed by SDS-PAGE and recombinant protein-containing aliquots were extensively dialyzed against PBS (137 mM NaCl, 10 mM Na_2_HPO_4_, 2.7 mM KCl and 1.8 mM KH_2_PO_4_, pH 7.4). Purified proteins were mixed with 12.5% Alhydrogel [2% Al(OH)_3_] (Brenntag Biosector) and used to immunize BALB/c mice for polyclonal serum obtainment.

**Table 1.** Oligonucleotides employed in this work.

Oligonucleotide	Sequence	Restriction enzyme
LIC11711 F	5ʹ- GGATCCTGTTTTACTACAAAAAGTACAGATAACG-3ʹ	BamHI
LIC11711 R	5ʹ- CCTACCTTATTTTCTGCGAATCACTTC-3ʹ	KpnI
LIC12587 F	5ʹ- GGATCCTGTACTAGCAGTCAGAAAACAGTGG-3ʹ	BamHI
LIC12587 R	5ʹ- CCTACCTTATCTTGGAAGAACCTGAGAAAG-3ʹ	KpnI
qLIC11711 F	5ʹ- AAAGGGGGAGACGTTTTGAT-3ʹ	–
qLIC11711 R	5ʹ- CGTTTCAAATGCTCCCGTAT-3ʹ	–
qLIC12587 F	5ʹ- CTTGGCTCTGGGCATTTTAG-3ʹ	–
qLIC12587 R	5ʹ- AGCCCTTCTTCCAGAACCAT-3ʹ	–
16S F	5ʹ- GGTGCAAGCGTTGTTCGG-3ʹ	–
16S R	5ʹ- GATATCTACGCATTTCACCGC-3ʹ	–

### Circular dichroism spectroscopy

Recombinant proteins were dialyzed against 0.1 M sodium phosphate buffer, pH 7.4 at 4°C. Spectroscopy analysis were measured using a 1 mm optical path cuvette at 0.5 nm/s intervals at 20°C and captured on a Jasco J-810 (Japan Spectroscopic) spectropolarimeter associated with a Peltier unit for digital temperature control. The spectra expressed in terms of residual molar ellipticity were submitted to analysis in BeStSel webserver for protein secondary structure contents (http://bestsel.elte.hu/index.php) [30,31]. *In silico* analysis of protein prediction was performed in PSIPRED webserver (http://bioinf.cs.ucl.ac.uk/psipred//) [32,33].

### Antiserum production in mice against recombinant proteins

Female BALB/c mice (4–6 weeks old) were immunized subcutaneously with 10 µg of each recombinant protein adsorbed in 10% (vol/vol) of Alhydrogel [2% Al(OH)3; Brenntag Biosector], used as adjuvant. Two subsequent booster injections were given at 2-week intervals with the same preparation. Negative control mice were injected with PBS/adjuvant. Two weeks after each immunization, mice were bled from retro-orbital plexus and pooled sera were analyzed by enzyme-linked immunosorbent assay (ELISA) for determination of antibody titers. Prior to experiments, anti-recombinant protein sera were adsorbed to a suspension of *E. coli* to suppress the reactivity of anti-*E. coli* antibodies [34].

### RNA extraction and real-time reverse transcriptase quantitative PCR (RT-qPCR)

Leptospiral cells were recovered from liquid EMJH culture medium by centrifugation (3,075 × *g*, 15 min, 4°C) and total RNA was extracted using TRIzol reagent (15596026, Invitrogen), as recommended by the manufacturer. Residual DNA was eliminated by incubation with DNAseI (0.1 U/µL, 18068-015, Invitrogen) for 60 min at room temperature and the cDNAs were obtained after reverse transcriptase PCR amplification of RNAs using SuperScript III kit Reverse Transcriptase (Invitrogen). RT-qPCR was performed using CFX96 Real-Time System (Bio-Rad) to detect synthesized double strand DNAs, using oligonucleotide pairs described in Table 1. Reactions were performed with SYBR Green PCR Master Mix (4309155, Applied Biosystems) in a 20 µL reaction volume. The cycle parameters were programmed for 95°C for 10 min; 40 cycles of 95°C for 15 s and 58°C for 1 min. The relative gene expression among leptospiral strains was performed using comparative 2^−ΔΔCT^ [35] and normalized with internal control 16S rRNA gene of *L. interrogans*.

### Immunoblotting assay

Recombinant proteins were transferred into a nitrocellulose membrane after separation by SDS-PAGE. The membrane was blocked with PBS solution and 0.05% Tween 20 (PBS-T) containing 10% skimmed dry milk (PBS-T/milk) at 4°C overnight. After PBS-T washes, the membrane was incubated for 1 h at 37°C with anti-His monoclonal antibody (anti-His MAbs; 1:10,000; A7058, Sigma-Aldrich) or polyclonal antiserum raised against rLIC11711 (1:400) or rLIC12587 (1:200), followed by incubation with horseradish peroxidase (HRP)-conjugated anti-mouse IgG (1:5,000; A9044, Sigma-Aldrich). Detection was revealed with Super Signal West Dura Extended Duration Substrate (34075, Thermo Fisher).

### *Evaluation of native proteins conservation among* leptospira *spp. by immunoblotting*

Bacterial cells were washed; resuspended in PBS and samples were standardized based on the absorbance at 420 nm. Immunoblotting of whole cell lysate extracts was performed as previously described [36], with incubation of anti-recombinant protein antisera (concentrations as described above) and HRP-conjugated anti-mouse IgG (1:5,000) (Sigma-Aldrich).

### *Protein localization in* L. interrogans *cells*

**(i) Immunofluorescence**. This assay was performed as described by Santos et al. [29]. Briefly, *L. interrogans* serovar Copenhageni strain M20 were harvested from culture media (3,075 × *g*, 15 min), resuspended in 2% paraformaldehyde solution (30°C, 40 min) and then incubated with 5% BSA in PBS solution. Leptospires were treated with antiserum against each recombinant protein, followed by incubation with propidium iodide (PI, P4864, Sigma-Aldrich) and anti-mouse IgG antibody conjugated with fluorescein isothiocyanate (FITC, F9006, Sigma-Aldrich). Non-immune serum and LipL46 (outer membrane protein) [37] were used as negative and positive control, respectively. Images were captured on a confocal immunofluorescence microscope of the LSM 510 META model (Zeiss, Oberkochen, Germany).

**(ii) Intact and lysed bacterial cells**. Live *L. interrogans* serovar Copenhageni strain M20 or *L. biflexa* serovar Patoc strain Patoc 1 cells in PBS solution were coated onto enzyme-linked immunosorbent assay (ELISA) plate (10^7^ cells/well) for 16 h incubation. After washing, wells were blocked with PBS containing 1% BSA. Plates were incubated with antisera against rLIC11711, rLIC12587 or the inner-membrane control protein LipL31 [38] for 1 h at 28°C. Wells were washed three times with PBS and incubated with 100 μL of HRP-conjugated anti-mouse IgG (1:5,000). The reactions were carried on, as previously described [14]. For statistical analysis, complete system for LIC11711 or LIC12587 was compared with LipL31 in each group by Student’s t-test. In other assay, ELISA plate was coated with intact or lysed *L. interrogans* cells and native proteins LIC11711, LIC12587 and LipL31 (control) were detected as described above. For statistical analysis, OD_492nm_ of lysed bacteria as considered 100% and compared to the signal obtained with intact bacteria for each protein by Student’s t-test.

**(iii) Proteinase K accessibility**. *L. interrogans* serovar Copenhageni strain M20 cells were resuspended in 15 mL of proteolysis buffer (10 mM Tris-HCl pH 8.0, 5 mM CaCl_2_) at the final concentration of 10^8^ cells/mL. Proteinase K (PK) (25 μg/mL) was added in each aliquot and enzyme activity was stopped by adding 100 mM PMSF after 0, 1 and 3 h. Bacteria were recovered and resuspended in 100 μL of PBS. Proteins of interest were visualized by western blotting using polyclonal antiserum (1:200) and HRP-conjugated anti-mouse IgG (1:5,000). The cytoplasmic protein DnaK [39], employed as negative control, was detected with polyclonal anti-DnaK antiserum (1:200) raised in mice. The relative densitometry of LIC11711 monomer and dimer and DnaK after 1 and 3 h incubation with PK was calculated in percentage comparing with non-treated sample (0 h).

### Reactivity of recombinant proteins with serum samples of patients diagnosed with leptospirosis and other unrelated diseases

Recombinant proteins (500 ng/well) were immobilized into ELISA plates, which were washed with PBS-T and blocked with PBS-T/milk for 2 h at 37°C. Leptospirosis patient’s serum samples at the onset (MAT-, n = 20) or convalescent phase (MAT+, n = 20) were diluted (1:100) in PBS-T/milk and then incubated for 1 h at room temperature. Specificity was evaluated by employing serum samples of patients diagnosed with dengue (n = 10), Chagas disease (n = 10) malaria (n = 10) and HIV (n = 10). Reactivity was assessed using HRP-conjugated anti-human IgG antibody (1:5,000, Sigma-Aldrich). Reaction was detected by adding 1 mg/mL *o*-phenylenediamine dihydrochloride (OPD, P8287, Sigma-Aldrich) in citrate phosphate buffer (pH 5.0) plus 1 μL/mL H_2_O_2_. The reaction carried out for 10 min and was stopped by the addition of 50 μL 2 M H_2_SO_4_. The serum samples used in this experiment were previously treated with 10% *E. coli* whole cell lysate in PBS-T/milk solution in order to avoid reactivity with anti-*E. coli* antibodies, as described previously [34]. The cut-off was calculated based on the mean absorbance obtained with commercial normal human serum (NHS, A3912, Sigma-Aldrich) plus three times the standard deviation (SD) between the same samples [40]. The samples with values above the cut-off were considered positive for this experiment.

### Binding of rLIC11711 and rLIC12587 to host components

One µg of immobilized component in ELISA plate wells was allowed to interact with 1 µg of recombinant protein, for 2 h at 37°C. Component-bound proteins were detected by incubation with mice polyclonal antisera (1:4,000 anti-rLIC11711 and 1:2,000 anti-rLIC12587) followed by HRP-conjugated anti-mouse IgG (1:5,000) or anti-His MAbs (1:10,000). Negative controls BSA and fetuin were included in the experiments. For statistical analysis, the mean absorbance values of each component were compared with the mean of the negative controls by Student’s t-test, and the p-value below 0.05 was considered statistically significant.

The statistically significant interactions were further characterized by dose-response assays; briefly, 1 µg of each host component or negative control BSA was coated onto ELISA plates and then increasing concentrations of recombinant proteins were added. The protein-component interaction was detected as described above. Data were plotted and analyzed in GraphPad Prism 6 to calculate the dissociation constant (K_D_), using a nonlinear regression fitting.

Vitronectin (250 ng/well) was immobilized in ELISA plates and incubated at 4°C overnight. Then, 5 μM recombinant protein plus different concentrations of heparin (0–100 mg/mL) were added and the interaction was maintained for 2 h at 37°C. Bound recombinant proteins were detected by incubation with HRP-conjugated anti-His MAbs and reactivity was developed by adding OPD solution, as described above.

### Characterization of recombinant proteins binding to PLG and PLA formation

Recombinant proteins interaction with PLG was also confirmed by far-western blotting, where membrane-transferred recombinant protein or BSA (experimental control) was allowed to interact with PLG solution (1 mg/mL) and bound component detected with polyclonal anti-PLG antibodies (1:5,000). The inverse interaction was also assayed: PLG was transferred to nitrocellulose membrane and recombinant proteins were added (5 µM solution) for interaction. Bound proteins were detected by incubation with HRP-conjugated anti-His MAbs. ELISA plate wells were coated with 1 µg PLG and incubated with 1 µg recombinant protein for different time (5, 15, 30, 60 and 120 min). The interaction was detected by incubating with anti-His MAbs. The participation of lysine residues in the recombinant protein-PLG interaction [41] was assessed by adding increasing concentrations (0 to 20 mM) of 6-aminocaproic acid (ACA, A2504, Sigma-Aldrich) to the interactions. To investigate the effect of ionic strength in the interaction between the proteins and PLG, the reaction was performed with increasing NaCl concentration (100 to 300 mM). The PLA formation from recombinant protein-bound PLG was performed as described by Vieira and colleagues [42].

### Inhibition of fibrin clot formation by recombinant proteins

This assay was performed essentially as described in Oliveira et al. [13]. In brief, different concentrations of recombinant protein (2.5–10 μM) were incubated with human Fg (1 mg/mL) for 2 h at 37°C under gentle agitation. Ninety microliters of the mixture were plated on ELISA together with 10 μL of thrombin solution (final concentration 0.2 U/mL) for fibrin clot formation; the reaction was monitored in triplicate by increased turbidity at the wavelength of 600 nm for 1 h with 2 min interval.

### Binding assay of the recombinant proteins to components of the complement system from normal human serum (NHS)

Recombinant protein immobilized on ELISA plates (1 μg/well) was incubated with solutions containing 5%, 10%, or 20% commercial NHS (Sigma-Aldrich) in PBS-T/BSA, for 2 h at 37°C. In sequence, goat anti-component IgG antibody was added (1:10,000) followed by incubation with HRP-conjugated anti-goat IgG (1:50,000) (A5420, Sigma-Aldrich). As negative control, NHS solution was incubated with immobilized BSA.

### *Protective effect of recombinant proteins upon* E. coli *survival after NHS challenge*

*E. coli* cells (10 µL of an OD_600_ = 0.4 solution) were incubated for 2 h at 37°C with 200 µL solution of 20% NHS, alone or previously incubated (2 h at 37°C) with recombinant proteins (0.5 or 2 µg). Inactivated NHS (iNHS; 56°C, 20 min) and PBS were used as controls. Cells were plated onto LB solid medium and incubated overnight at 37°C. Plates were equally divided into eight parts and cells were counted in two parts per plate; the values were multiplied by four to obtain the number of colonies recovered after challenge with NHS. For statistical analysis, three independent experiments were performed in triplicate for each treatment, and number of colonies was compared to those obtained after challenge with only NHS treatment (minimal survival), PBS and iNHS (maximal survival), by two-tailed t-student test.

### *Incubation of* L. interrogans serovar *Copenhageni strain M20 with serum against each recombinant protein prior to NHS challenge*

Leptospiral cells (10^8^ cells/treatment) were harvested from the culture medium (3,075 × *g*, 15 min), washed twice in PBS and then incubated with heat-inactivated mouse polyclonal antiserum of each recombinant protein (1:100 dilution in PBS) for 1 h at room temperature. Pre-immune mouse serum was employed as control at the same dilution. Then, cells were recovered by centrifugation and challenged with 20% NHS or iNHS in PBS. After 1 h incubation at 30°C, solutions were diluted 1:10 and visualized in Petroff-Hausser counting chamber for viable and motile cells counting. For each group, leptospires counting after iNHS treatment was considered 100% of cell viability. As a control, the anti-leptospiral proteins sera were heated at 56°C and checked for their ability to react with their respective antigen.

### Ethics statement

All animal studies were approved by the Ethical Committee for Animal Research of the Instituto Butantan, Brazil, registered under protocol no. 6,819,120,116. The Committee for Animal Research in Instituto Butantan adopts the guidelines of the Brazilian College of Animal Experimentation (COBEA). This work was also analyzed and approved by Ethics Committee on Human Research of the Instituto de Ciências Biomédicas (CEPSH) of the Universidade de São Paulo, São Paulo, Brazil, which certificated that the work does not involve human manipulation to warrant approval, as the ethical principles required by the Committee under the protocol no. 806/2016.

## Results

### In silico *analysis of LIC11711 and LIC12587 coding sequences localization*

Proteins were selected based on their predicted cellular localization in *L. interrogans*, since outer membrane proteins (OMPs) are likely to be involved in host–pathogen interactions. LIC11711 and LIC12587 genes were identified in chromosome I from *L. interrogans* serovar Copenhageni. LIC11711 protein was predicted to be periplasmic/outer membrane and outer membrane by PSORT [25] and CELLO [24], respectively, while LIC12587 was predicted to be an outer/cytoplasmic membrane. LipoP analysis predicted both proteins as lipoproteins, comprising an N-terminal sequence recognized by SpII (signal peptidase II), responsible for cleavage and covalent attachment of a fatty acid in cysteine, thus, allowing membrane anchorage [26]. BLASTp analyses [27] of the coding sequences LIC11711 and LIC12587 show that these sequences present low percentage of coverage and identity with other bacterial genuses.

### Cloning of LIC11711 and LIC12587 cdss and recombinant protein expression

**Figure 1.**
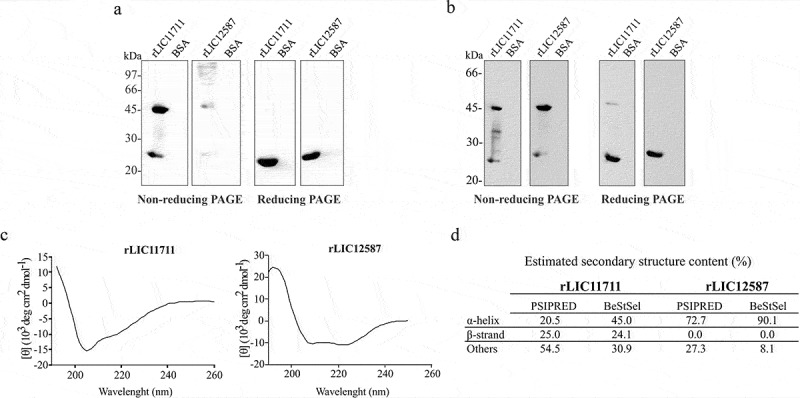
Recombinant protein expression, western blotting and structure evaluation. Analysis of recombinant proteins by western blotting under non-reducing or reducing treatment (β-mercaptoethanol and heating at 96°C). Protein bands were detected after monoclonal (MAbs) anti-His incubation (1:10,000) (a) and polyclonal anti-rLIC11711 (1:400) or anti-rLIC1212587 (1:200) (b). BSA was employed as control. In (c) Secondary structure evaluation of rLIC11711 and rLCI12587 by circular dichroism. The spectra were obtained in a spectropolarimeter using a 1 mm optical path cell with 0.5 nm intervals. (d) Amino acids sequences were submitted to PSIPRED webserver and CD spectra data were submitted to BeStSel webserver for prediction and experimental secondary structure content of both recombinant proteins.

The coding sequences of LIC11711 and LIC12587 without the signal peptide sequences were cloned into pAE vector and the recombinant plasmids used to transform *E. coli* BL21 (DE3) Star pLysS strains. The recombinant proteins rLIC11711 (22.8 kDa) and rLIC12587 (23.9 kDa) were expressed with 6xHis tag at the N-terminal portion, and purified by affinity chromatography (Ni^2+^) from the soluble fraction of the bacterial cell lysate (data not shown). Recombinant protein expression was confirmed by western blotting, probed with anti-His MAbs (Figure 1(a)). Interestingly, two bands could be clearly observed for rLIC11711 protein under non-reducing conditions (Figure 1(a)). As the monomeric state of this recombinant protein is predicted to be 22.8 kDa, the higher molecular mass band of approximately 46 kDa represents, most probably, the dimeric state of the protein. This form of protein was not observed under reducing condition (Figure 1(a)). The rLIC12587 protein, when probed with anti-His MAbs, showed several higher molecular mass bands and two other weak bands of approximately 24 kDa, and 48 kDa (Figure 1(a)) under non-reducing conditions, contrasting to a single band under reducing conditions (Figure 1(a)). No reactivity was observed with the samples in which BSA was used as a control protein (Figure 1(a)). Possibly, the higher molecular mass bands detected with rLIC12587 under non-reducing conditions are not specific, probably due to contaminants co-purified with the protein. The dimer and monomer were confirmed when the blotted protein was probed with anti-rLIC11711 or anti-rLIC12587 (Figure 1(b)).

The structural conformation of recombinant proteins was also evaluated by circular dichroism spectroscopy, which provided protein secondary structure (Figure 1(c)). CD spectra data were submitted to BeStSel webserver and the percentage of secondary structure content of each protein is depicted in Figure 1(d). The data show a mixture of secondary structure contents for rLIC11711, while predominance in α-helix content is detected in rLIC12587. For comparative purpose, the percentage of *in silico* analysis for secondary structure prediction is also shown in this figure. The comparison between predicted and experimental secondary structures for each protein shows comparable tendency, mixture of content for rLIC11711 and α-helix predominance for rLIC12587 (Figure 1(d)).

### *Conservation of LIC11711 and LIC12587 among* leptospira *spp.*

**Figure 2.**
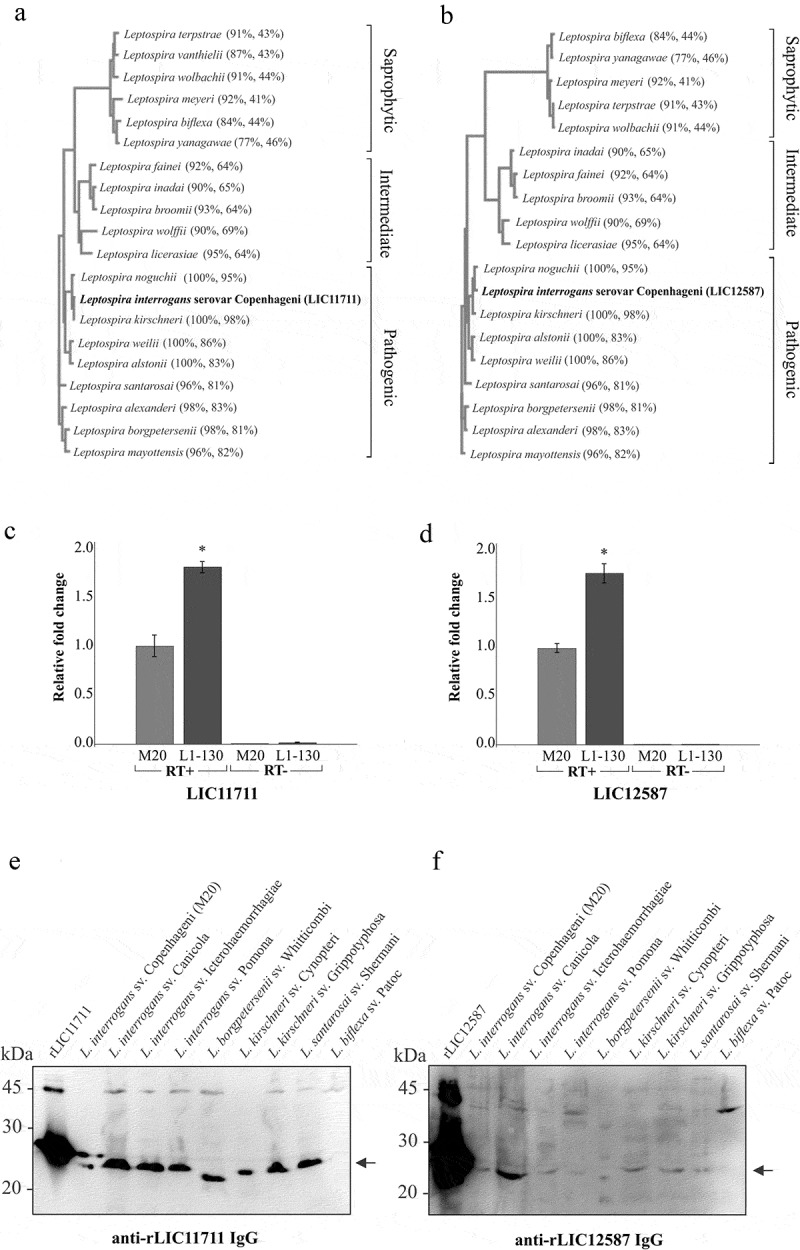
Analysis of protein conservation among *Leptospira* strains *in silico*, RT-qPCR and western blotting. Amino acids sequences from proteins encoded by LIC11711 (a) and LIC12587 (b) genes were analyzed *in silico* by BLASTp with sequences available in the GenBank database and were used to perform multiple alignments on Clustal Omega. The phylograms generated show a high degree of identity between pathogenic species, whereas the saprophytic species presented a lower conservation of the target sequences. The numbers in parentheses show the percentage of coverage of the database sequences in relation to the query sequence and the percentage of identity between the database sequences with the query sequence per length of coverage. Relative gene expression comparison of LIC11711 (c) and LIC12587 (d) in virulent L1-130 and in culture-attenuated M20 strains of *L. interrogans* serovar Copenhageni by RT-qPCR. Amplification was performed with reverse transcriptase (RT+) and in the absence of enzyme (RT-), as experimental control. The gene transcription was calculated using 2^−ΔΔCT^ method and normalized with 16S rRNA. Representative results refer to one independent experiment out of two and mean and error bars were calculated based on experimental triplicate. Statistical analyses were performed comparing relative gene expression of both strains by Student’s t-test (*p < 0.05). Protein conservation was also performed using whole cell leptospiral extracts by western blotting. Native proteins were detected by anti-LIC11711 (e) and anti-LIC12587 (f) and HRP-conjugated mouse anti-mouse IgG secondary antibody. *L. interrogans* serovar Copenhageni strain used is M20.

A comparison of the CDSs performed by BLASTp revealed a high amino acid sequence identity of the proteins LIC11711 (Figure 2(a)) and LIC12587 (Figure 2(b)) of *L. interrogans* serovar Copenhageni strain L1-130 with other pathogenic species of *Leptospira*, whereas lower identity was observed with the intermediate and saprophytic species, including *L. biflexa* [43].

Gene expression levels in virulent and culture-attenuated pathogenic *L. interrogans* serovar Copenhageni were compared by RT-qPCR (Figure2(c and d)). The results revealed the presence of LIC11711 and LIC12587 mRNAs in both strains, with higher transcription expression in virulent L1-130 compared to the culture-attenuated strain, of approximately 1.82 and 1.76 fold-change for LIC11711 and LIC12586 mRNAs, respectively.

The conservation of the proteins was experimentally evaluated by western blotting after probing leptospiral cell extracts with polyclonal serum anti-recombinant proteins. The presence of ~23 kDa LIC11711 (Figure 2(e)) and ~25 kDa LIC12587 (Figure 2(f)) was detected only in the pathogenic *Leptospira* species, confirming the conservation of proteins in different pathogenic species of *Leptospira*. Moreover, it was not possible to observe bands related to either protein in saprophytic *L. biflexa* cell extract, while a higher molecular mass band was observed in almost all cellular extracts, most probably due to non-specific reaction.

The detection of transcripts and proteins referring to the studied genes corroborates the study published by Malmström and colleagues [20], in which both proteins were detected and quantified in whole leptospiral extract by mass spectrometry.

### *Native proteins localization in* L. interrogans *by ELISA*

**Figure 3.**
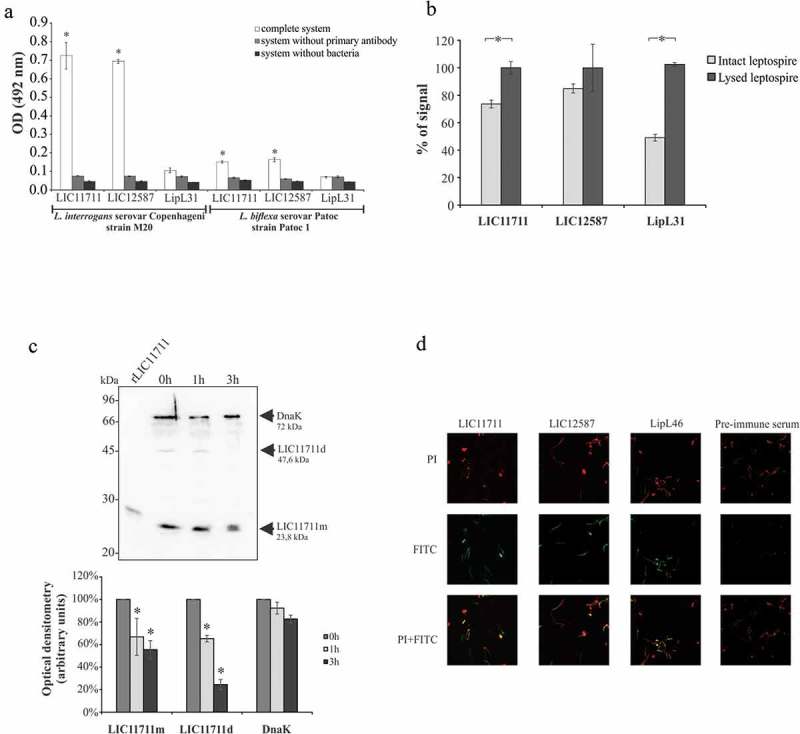
Cellular localization of native proteins in *L. interrogans* serovar Copenhageni. Evaluation of native proteins in intact (a) or lysed (b) *L. interrogans* serovar Copenhageni strain M20 by ELISA. Leptospires were immobilized on 96-well plates and incubated with polyclonal antiserum against recombinant protein followed by HRP-conjugated anti-mouse antibody addiction. *L. biflexa* was used as a control to differentiate pathogenic leptospires from non-pathogenic (a) and the cytoplasmic protein LipL31 was used as a parameter to differentiate surface exposed from cytoplasmic proteins. Statistical analyses were calculated comparing complete system with LipL31 in each group by Student’s t-test (*p < 0.05) (a). In (b) OD_492nm_ values obtained in each group for the lysate treatment were considered 100% of signal and was compared to the signal obtained with the intact bacteria by Student’s t-test (*p < 0.05). (c) Protein accessibility by proteinase K degradation. Viable *L. interrogans* serovar Copenhageni (strain M20) cells were incubated with 25 μg/ml PK for 1 and 3 h. Cells were heated and cellular extracts were transferred to nitrocellulose membrane. For protein detection, membrane was incubated with polyclonal antiserum against rLIC11711 or rLIC12587 and DnaK (experimental control) followed by HRP-conjugated anti-mouse antibody (upper panel). Protein degradation after 1 and 3 h incubation with PK was measured by band densitometry and statistical analysis were calculated comparing with non-treated sample (0 h) by Student’s t-test (*p < 0.05). Representative results refer to one independent experiment out of two. m: monomer; d: dimer. (d) In immunofluorescence assay, *L. interrogans* serovar Copenhageni strain M20 were fixed and treated with the antiserum against rLIC11711, rLIC12587, LipL46 (positive control) or pre-immune serum (negative control). Leptospiral DNA was labeled by propidium iodide (PI). The co-localization was performed by overlapping the images of leptospires marked by PI and the target proteins labeled by fluorescein isothiocyanate (FITC).

The localization of native proteins in intact *L. interrogans* was verified by ELISA (Figure 3(a)). The results suggest the detection of the native proteins expressed by the LIC11711 and LIC12587 genes in *L. interrogans* M20 strain only with the complete system (containing leptospires, primary and secondary antibodies), whereas no significant difference was observed in the assays where the primary antibodies or bacteria were omitted. The cytoplasmic protein LipL31 [38] was used as control, and accordingly, no reactivity was detected. For *L. biflexa* serovar Patoc, the values of the complete system were much lower when compared to the strain *L. interrogans* M20 for both proteins. These interactions of lower intensity may be due to the lower identity of both proteins in the saprophytic *L. biflexa* serovar Patoc strain of 44% and 31% for LIC11711 and LIC12587, respectively.

The results obtained using intact and lysed bacterial cells (Figure 3(b)), showed an increase of 53% on the signal of the cytoplasmic LipL31 after cell lysis, followed by 25% and 14% increase for LIC11711 and LIC12587, respectively.

We have also examined treatment of intact leptospires with proteinase K, followed by protein detection with antibodies [44,45]. Leptospires were treated with proteinase K, and aliquots of the reaction mixture were taken at 0, 1 and 3 h incubation. Samples were gel fractionated, proteins blotted into the membranes, and then probed with antiserum against anti-LIC11711 and anti-DnaK (Figure 3(c) – upper panel). Both forms of the protein, monomer (LIC11711m) and dimer (LIC11711d) were susceptible to protease treatment, as protein bands gradually disappear in function of time, as detected by optical density measurements (Figure 3(c)-lower panel). Similar assay with LIC12587 was not possible to perform due to the difficulty in detecting this protein in leptospiral cells (compare Figure 2(e with f)).

### *Protein localization in* leptospira *by immunofluorescence assay*

Paraformaldehyde-fixed leptospires were incubated with antiserum produced against the corresponding recombinant protein and native proteins were detected with FITC-conjugated anti-IgG antibody (Figure 3(d)). Green fluorescence signal was observed with both proteins and the positive control LipL46, suggesting that the proteins were FITC-labeled. In addition, co-localization of the proteins leptospires was confirmed through the overlap of the FITC and PI marking images. Only PI fluorescence was detected for the leptospires treated with pre-immune antiserum. Taken together, our results suggest that these proteins are surface-exposed, and therefore interesting targets for host interactions.

Taking together, the results shown in Figure 3 from different assays suggest that these proteins are surface-exposed in *Leptospira*. Moreover, because under non-reducing, more physiological conditions, the recombinant proteins have a quaternary structure of dimer (rLIC11711) and oligomer (rLIC12587), it is possible that their assembly when anchored to the membrane might be distinctive.

### Reactivity of recombinant proteins with serum samples of confirmed leptospirosis and of unrelated diseases

**Figure 4.**
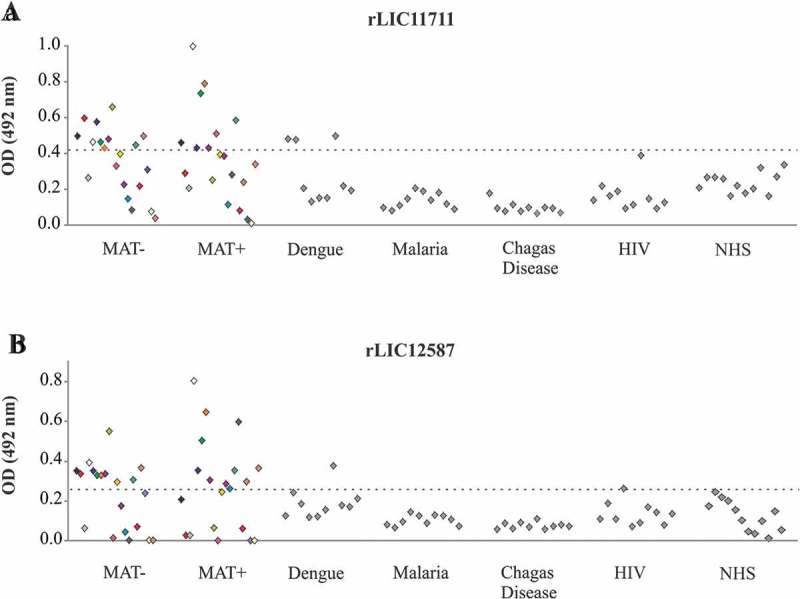
Reactivity of recombinant proteins with serum samples of confirmed leptospirosis and of other unrelated diseases. Recombinant proteins (a) rLIC11711 and (b) rLIC12587 were immobilized on 96 well plates (250 ng/well). Reactivity was assessed by total IgG antibodies in human paired sera collected from patients at the onset (MAT-) and convalescent phase (MAT+), depicted by the same color, detected by incubation with HRP-conjugated anti-human IgG antibodies (1:5000). The cutoff values represented by the horizontal dashed line were defined as the mean plus three SD of the absorbance values from normal human serum (NHS).

We evaluated the reactivity of these proteins with paired-serum of 20 confirmed leptospirosis samples, corresponding to the onset (MAT-) and convalescent phase (MAT+) of the disease. Figure 4(a) shows a reactivity of 50% of MAT-negative sera and 40% of MAT-positive sera for rLIC11711, while rLIC12587 presented 55% reactivity for both MAT-negative and positive samples (Figure 4(b)). Almost no reactivity was observed when proteins were incubated with serum samples of unrelated diseases, 100% specificity of both recombinant proteins against malaria and Chagas disease, 70% specificity of rLIC11711 against dengue, and 90% specificity of rLIC12587 against dengue and HIV (Figure 4(a and b)). The detection of anti-LIC11711 and anti-LIC12587 in serum samples of confirmed leptospirosis patients shows that both proteins are expressed by pathogenic leptospires during infection and could be used for development of diagnostic assays.

### Assessment of ECM host molecules as targets for rLIC11711 and rLIC12587 attachment

**Figure 5.**
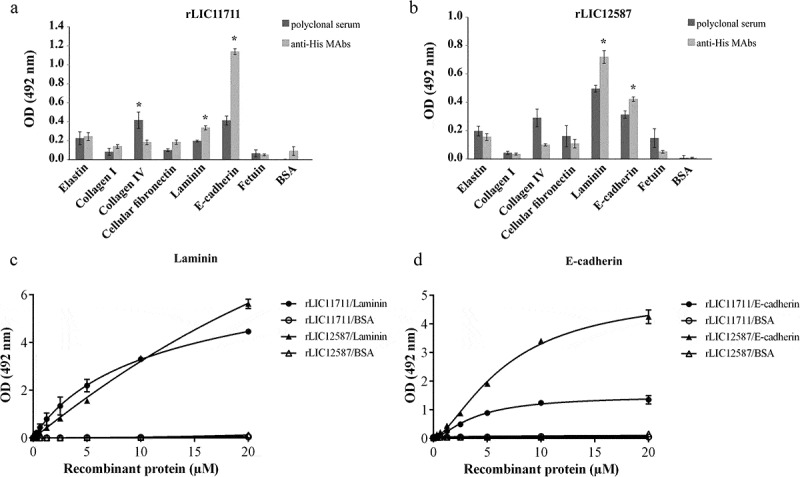
Binding of recombinant proteins with select human host molecules. ELISA plates were coated with ECM components and negative control fetuin and BSA. Recombinant proteins (a) rLIC11711 or (b) rLIC12587 was added and bounded proteins were detected by incubation with anti-His MAbs or polyclonal antiserum against each protein, plus HRP-conjugated anti-mouse IgG; the reactions were developed using the HRP substrate OPD. Representative results refer to one independent experiment out of two and bars represent the mean ± SD absorbance at 492 nm of experimental triplicates. Statistical analyses were calculated comparing with negative controls by Student’s t-test (* p < 0.05). Dose-response assays were performed with recombinant proteins and ECM components laminin (c) and E-cadherin (d). ELISA plates were coated in triplicate with ECM components or negative control BSA, blocked and then incubated with increasing concentrations of recombinant protein (0–20 µM). Binding detection was performed by incubating monoclonal HRP-conjugated anti-His antibody (1:10,000) and developed by the addition of the OPD substrate. Each point of the curve was performed in triplicate and the value was expressed as the mean ± SD.

Since our results strongly suggest that both proteins are surface-exposed, we set out to examine the ability of recombinant proteins to interact with the host components, laminin, collagen I and IV, cellular fibronectin, elastin, E-cadherin components, as previously described [46]. Both recombinant proteins exhibited adhesion to laminin and E-cadherin, whereas rLIC11711 also showed binding to collagen IV (Figure 5(a and b), respectively). Similar results were obtained when interaction was performed with anti-His MAbs. Dose-response assay was performed in which each component was immobilized and incubated with increasing concentrations of each recombinant protein. The protein rLIC11711 showed a dose-dependent, non-saturable interaction with laminin (Figure 5(c)) and dose-dependence and saturable interaction with E-cadherin (Figure 5(d)); for the latter, saturation was reached at 10 μM of recombinant protein with a dissociation constant of (K_D_) of 3.82 ± 0.21 μM. A dose-dependence behavior between rLIC11711 and collagen IV was not observed (data not shown). For rLIC12587, both interactions with laminin and E-cadherin were dose-dependent and non-saturable.

### Adhesion of rLIC11711 and rLIC12587 to plasma components

**Figure 6.**
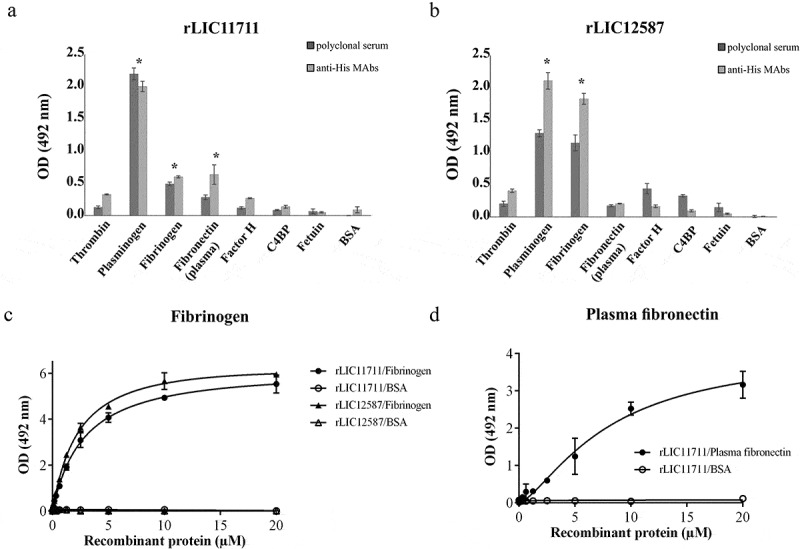
Binding of recombinant proteins with human plasma components. ELISA plates were coated with plasma components and negative controls fetuin and BSA. Recombinant proteins (a) rLIC11711 or (b) rLIC12587 was added and binding was measured using anti-His MAbs or polyclonal serum against each protein, plus HRP-conjugated anti-mouse IgG. Representative results refer to one independent experiment out of two and bars represent the mean ± SD absorbance at 492 nm of experimental triplicates. Statistical analyses were performed comparing with the negative controls by Student’s t-test (* p < 0.05). Dose-response assays were performed with recombinant proteins and plasma components Fg (c) and plasma fibronectin (d). ELISA plates were coated with these components or the negative control BSA and incubated with increasing concentrations of recombinant protein (0–20 µM). Each point of the curve was performed in triplicate and the value was expressed as the mean ± SD.

The ability of recombinant proteins to interact with plasma components thrombin, PLG, Fg, plasma fibronectin, and the complement regulators, factor H, and C4BP was evaluated by ELISA. Both recombinant proteins interacted with PLG and Fg, while only rLIC11711 reacted with plasma fibronectin, when the reactions were probed with either antiserum against each protein or anti-His MAbs (Figure 6(a and b)). The reactions of both proteins with Fg were dose dependent on protein concentration, but non-saturable (Figure 6(c)). When assayed, the proteins bound to Fg did not reduce fibrin clot formation, in a thrombin-catalyzed reaction (data not shown). Reactivity of protein rLIC12587 with plasma fibronectin was dose-dependent on protein concentration but saturation was not reached (Figure 6(d)).

### Evaluation of recombinant protein interactions with PLG

**Figure 7.**
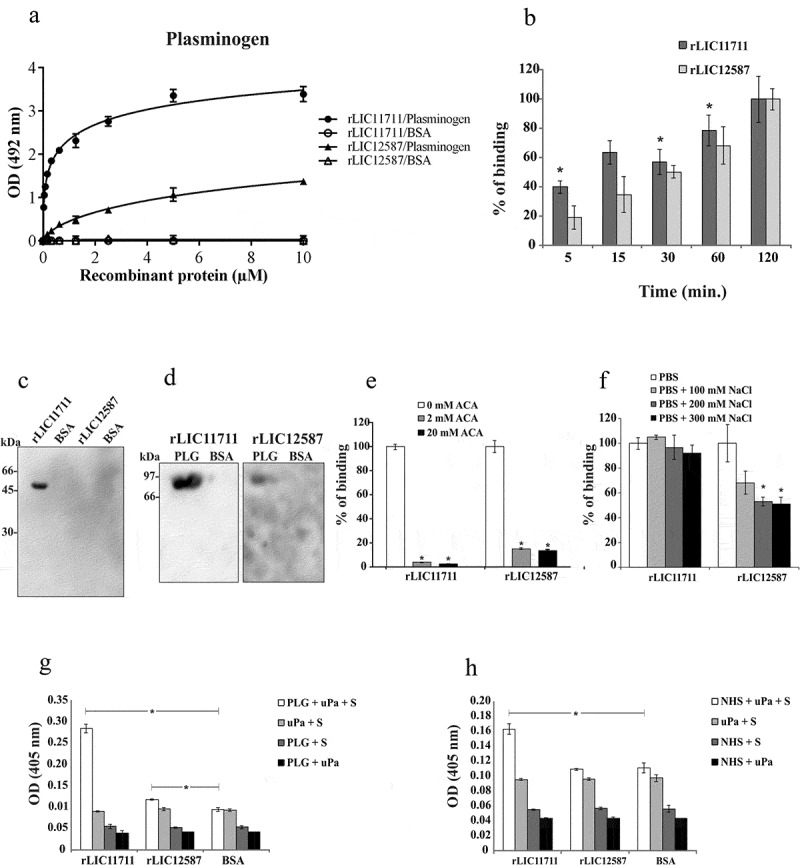
Characterization of recombinant protein interactions with PLG. Binding of recombinant proteins with PLG was evaluated by a dose-response assay using increasing concentrations of recombinant proteins interacting with coated PLG or BSA (a), or at different incubation times (b). Representative results refer to one independent experiment out of three and each point of the curve was performed in triplicate and the value was expressed as the mean ± SD absorbance at 492 nm for dose-response assay and percentage of binding ± SD for time-response assay was compared to the 120-min time point for the Student’s t-test. (c) Non-denatured recombinant proteins were transferred to membranes, which were blocked and incubated with PLG. Detection was performed by incubating the membrane with anti-PLG antibody followed by HRP-conjugated anti-mouse IgG antibody. (d) PLG was transferred to membrane after gel electrophoresis (non-reducing) and incubated with the recombinant proteins. Detection was performed by incubating the membrane with peroxidase-conjugated anti-His antibody. The involvement of kringle domains (e) or salt concentration (f) on PLG binding to recombinant proteins was verified by ELISA, using 2 and 20 mM of ACA (e), considering 0 mM ACA as 100% of ligation, and increasing concentration of NaCl (f). Representative result refers to an independent experiment out of three and mean and error bars were calculated based on experimental triplicate. (g and h) Production of PLA from PLG bound to recombinant proteins as evaluated by ELISA. Microplates coated with recombinant protein were incubated with purified PLG (g) or 30% NHS (h), plus the uPA and the chromogenic substrate D-val-leu-lys-p-nitroanilide dihydrochloride. For experimental controls, one of each component was omitted. Each assay was performed in triplicate and mean and error bars represents and absorbance’s (405 nm) were calculated from the mean ± SD. For the statistical analysis, the complete systems of rLIC11711 and rLIC12587 were compared with the BSA and evaluated by Student t-test (* p < 0.05).

The binding of rLIC11711 and rLIC12587 to PLG were dose-dependent but non-saturable (Figure 7(a)). The binding of both recombinant proteins with PLG was also evaluated in a time basis (Figure 7(b)), and an increase in binding was observed over time, being more prominent for rLIC11711 in which a 40% binding was observed in only 5 min compared to the maximal value obtained (2 h of interaction).

We examined whether the structural conformations of the expressed proteins are required for PLG binding, by western blotting. The presence of a band of approximately 45 kDa, corresponding to the rLIC11711 dimer, could be observed (Figure 7(c), lane 1), when the protein was blotted into the membrane, incubated with PLG and the reaction probed with anti-PLG, while no band for rLIC12587 (Figure 7(c), lane 3) or negative control BSA (lane 2 and 4) was detected. When PLG transferred to membrane was incubated with recombinant proteins and the reaction was probed with anti-His MAbs, a band of approximately 92 kDa, corresponding of PLG, was detected, especially for rLIC11711 (Figure 7(d), lane 1), followed by a modest reactivity for rLIC12587 (lane 3) and no reactivity for negative control BSA (lane 2 and 4). The data suggest that (i) the interaction between PLG and LIC11711 is stronger (higher affinity) or more stable than that between PLG and LIC12587 and (ii) the interaction between PLG and LIC11711 occurs with native PLG (Figure 7(c and d)).

It is known that kringle domains of PLG mediate the interaction with lysine residues of various bacterial proteins, including *Leptospira* [15,42,47]. We set out to investigate the role of these domains on the protein interactions using the lysine analog ACA. The results showed that the presence of ACA at a concentration of 2 mM almost totally inhibited the reaction of PLG with rLIC11711, while in the reaction with rLIC12587, a low percentage of binding remained, either with 2 or 20 mM (Figure 7(e)). Increasing NaCl concentration shows that electrostatic interaction is not important on the binding of PLG with rLIC11711, while an effect was detected with high NaCl concentration on the reaction of PLG and rLIC12587 (Figure 7(f)). The data suggest that ionic strength, under physiological condition, is not significant for the interaction of PLG and the recombinant proteins.

### Generation of PLA from PLG bound to recombinant proteins

The conversion of PLG to its enzymatically active form, PLA requires specific tissue-type (tPA) or urokinase-type (uPA) PLG activator [42]. The ability of recombinant protein-bound PLG to be converted to PLA, in the presence of uPA, was indirectly evaluated in the presence of PLA substrate. The results indicated that purified PLG bound with rLIC11711 could be converted to PLA, although in the case of rLIC12587, PLA generation, although statistically significant, is probably not relevant (Figure 7(g)). Controls of the reaction in which one of the components was omitted did not present enzymatic activity (Figure 7(g)). In order to evaluate whether the recombinant proteins were capable of capturing PLG from NHS and generate PLA, we replaced human purified PLG by 30% NHS solution. The results showed that rLIC11711, but not rLIC12587, has the ability to recruit PLG from NHS and generates PLA (Figure 7(h)). No reaction was observed when BSA was used as control protein.

These results suggest that the protein encoded by the LIC11711 gene has the potential to capture PLG from NHS, which may benefit the bacteria with the proteolytic activity of PLA, contributing to tissue invasion and immune system evasion.

### Interaction of rLIC11711 and rLIC12587 with complement system components

**Figure 8.**
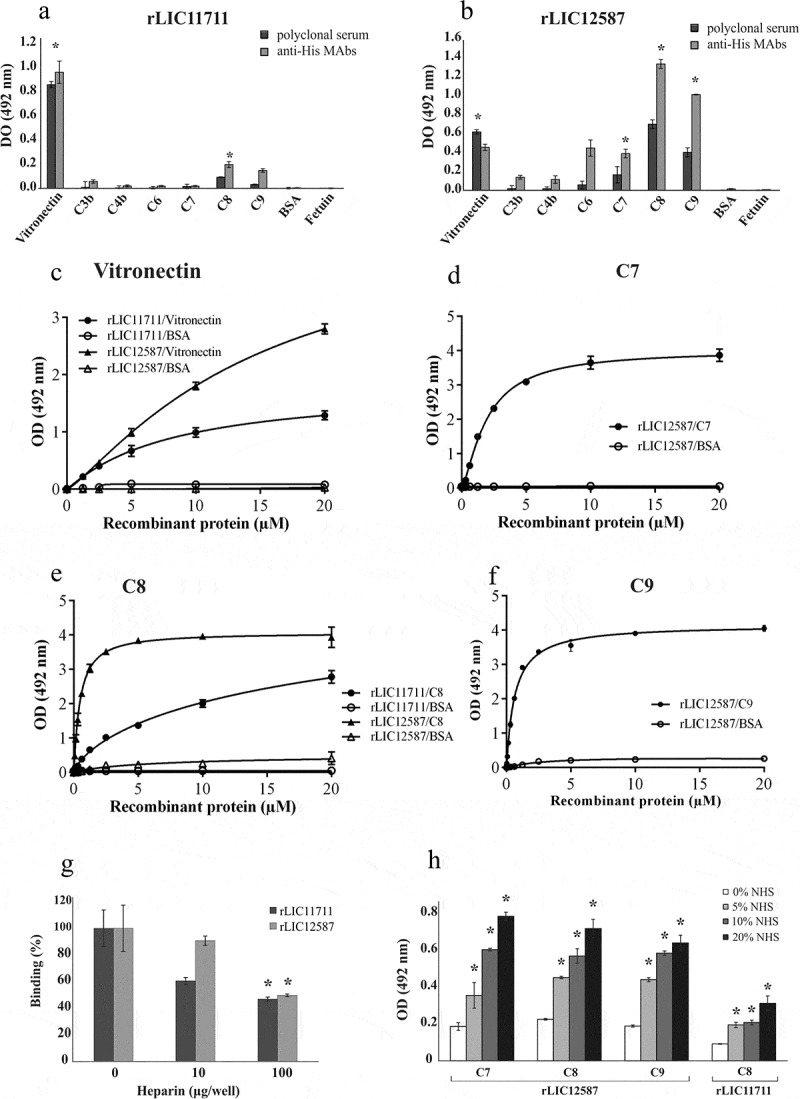
Binding of recombinant proteins with human complement system. The distinct components and the negative control proteins BSA and fetuin were adsorbed into the wells and incubated with 1 μg/well of (a) rLIC11711 or (b) rLIC12587. For binding detection, the anti-His MAbs or antiserum against recombinant protein plus HRP-conjugated anti-mouse were used. Representative results refer to one independent experiment out of two and bars represent mean ± SD of an experimental triplicate. For statistical analysis, the binding of recombinant proteins was compared to their binding to fetuin and BSA by Student’s t-test. For each statistically significant binding, a dose-response assay was performed with (c) vitronectin, (d) C7, (e) C8 and (f) C9. Components and negative control BSA were immobilized and increasing concentrations of recombinant protein were added to wells. Bound proteins were detected with anti-His mAbs. Each point of the curve represents a triplicate and the value was expressed as the mean ± SD. (g) The vitronectin was immobilized and incubated with the recombinant protein plus increasing amounts of heparin; binding was detected incubating anti-His mAbs. For statistical analysis, binding of recombinant proteins to vitronectin was compared to treatment without heparin by Student’s t-test. (h) Capture of complement components from NHS by recombinant proteins. Recombinant proteins were immobilized and incubated with increasing concentrations of NHS. The interaction with complement components was assessed by the addition of specific antibody against each component plus HRP-conjugated secondary antibody. Absorbance represents the mean ± SD of a triplicate. For statistical analysis, acquisition of complement by recombinant proteins was compared to treatment without NHS by Student’s t-test (*p < 0.05).

It has been reported the importance of surface proteins in mediating the evasion of the complement system and inhibition of membrane attack complex (MAC) formation by virulent *Leptospira*, but not by the saprophyte *L. biflexa* [16,48–50]. Thus, we decided to examine whether the proteins rLIC11711 and rLIC12587 had the ability to interact with the components of the human complement system. The rLIC11711 interacted in a dose-dependent and non-saturable manner to vitronectin and C8 (Figure 8(a, c, e)). The rLIC12587 showed a higher affinity interaction to components of the terminal complement pathway when compared to rLIC11711. Dose-response and saturable binding was observed with rLIC12587 and C7 (K_D_ = 1.95 ± 0.07 μM), C8 (K_D_ = 0.47 ± 0.01 μM) and C9 (K_D_ = 0.63 ± 0.06 μM) (Figure 8(d–f), respectively). The binding of rLIC12587 to vitronectin, similar to rLIC11711, was dose-dependent on protein concentration, but saturation was not reached (Figure 8(c)). The data from experiments showed in Figure 8(a–f) suggest that leptospiral LIC12587 might also interact with the complement pathway during an infection.

### Binding characterization of recombinant proteins with vitronectin

Based on the fact that the recombinant proteins rLIC11711 and rLIC12587 interact with vitronectin, we analyzed whether this binding occurs via heparin-binding domain. Vitronectin was immobilized on ELISA plate and incubated with recombinant proteins and increasing concentrations of heparin, used as a competitor by the heparin-binding domain. A reduction on the binding of recombinant proteins to vitronectin was observed, dependent on heparin concentration (Figure 8(g)). There was approximately a 40% reduction for rLIC11711 and 10% for rLIC12587 when 10 μg/well of heparin was used, and 54% and 51%, with 100 μg/well of heparin. Thus, the results suggest that both recombinant proteins interacted with vitronectin at least, partially, via this domain.

### Acquisition of components of the terminal complement pathway from normal human serum (NHS) by recombinant proteins

Immobilized recombinant proteins were incubated with normal human serum solution (5%, 10%, and 20%) and binding was detected using specific antibody against each component (Figure 8(h)). Higher interactions were observed with increasing concentration of NHS; rLIC12587 was capable of capturing C7, C8 and C9 and rLIC11711 of C8 (Figure 8(h)). The results indicate that both recombinant proteins are efficient in capturing the components in a more physiological condition.

### rLIC12587 and rLIC11711 proteins reduce the bactericidal effect of normal human serum

**Figure 9.**
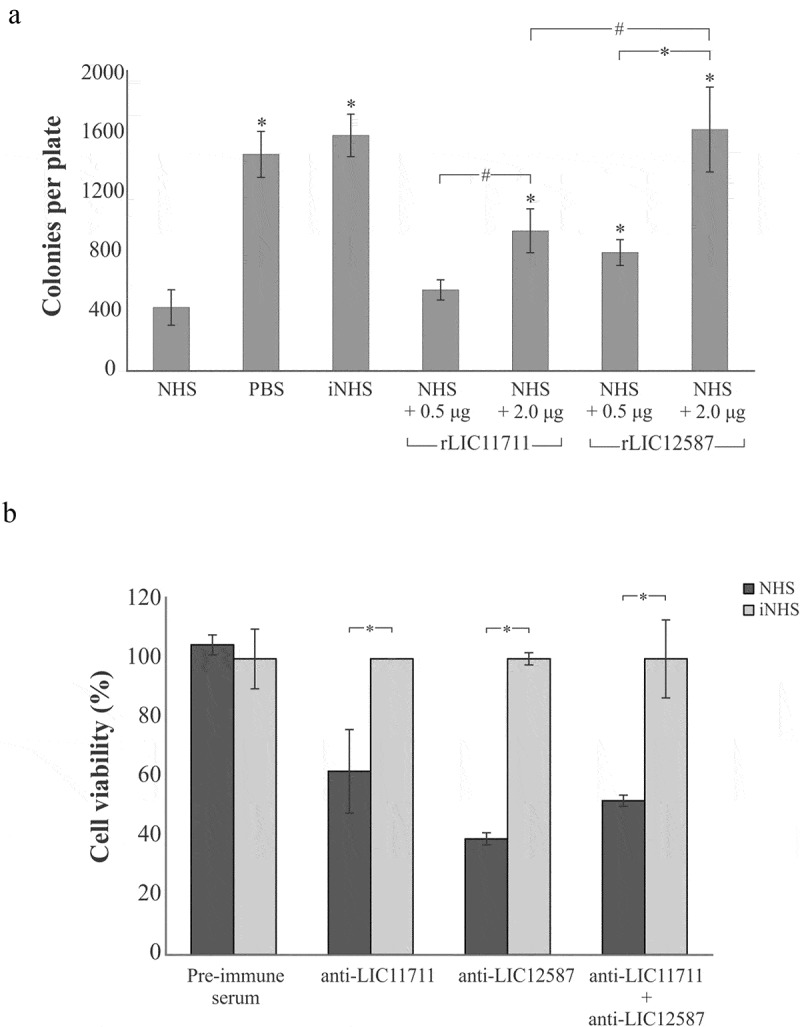
Effect promoted by (a) rLIC12587- or rLIC11711- treated with NHS in *E. coli* and (b) by anti-recombinant proteins sera in *L. interrogans* M20 strain prior to NHS exposure. (a) Solutions of 20% NHS in PBS were incubated with different quantities of rLIC12587 or rLIC11711 and the effect of the recombinant proteins on the capture of components from the serum complement system was evaluated by counting surviving *E. coli* DH5α colonies seeded on LB plates. For statistical analysis, the number of colonies per plate was compared with treatment with NHS only (*p < 0.05) and between 0.5 μg and 2 μg of the recombinant proteins (#p < 0.05) by Student’s t-test. (b) *L. interrogans* serovar Copenhageni strain M20 cells were incubated with heat-inactivated mouse polyclonal anti-each recombinant protein serum (1:100) and then challenged with 20% NHS or iNHS in PBS solution. Serum resistance was assessed by counting viable motile leptospiral cells in Petroff-Hausser chamber; for comparative purposes, leptospiral counting after iNHS treatment was considered 100% of cell viability. *p < 0.05. Representative result refers to one independent experiment out of two and mean and error bars were calculated based on experimental triplicate.

*E. coli* DH5α was used to determine the effect of the recombinant proteins upon the bactericidal activity of complement components present in NHS (Figure 9(a)). When incubated with saline or iNHS, *E. coli* cells were able to form 1,461 ± 154 and 1,589 ± 154 colonies per plate, respectively. Incubation with 20% NHS solution with no previous interaction with recombinant proteins culminated in a reduced number of colonies, 428 ± 119 per plate, demonstrating the lytic effect of serum upon *E. coli* cells. Prior incubation of NHS with 0.5 µg rLIC11711 resulted in 546 ± 68 colonies, while incubation with 2 µg the number of colonies recovered increased to 945 ± 148. For rLIC12587, we observed 798 ± 87 and 1,629 ± 286 colonies per plate after NHS incubation with 0.5 µg and 2 µg of protein, respectively. Although rLIC11711 showed a partial inhibition in serum bactericidal effect, we highlight the full protective effect of 2 µg rLIC12587 upon serum bactericidal activity. These results suggest that both proteins, more prominently LIC12587, might play a role in complement system evasion by pathogenic *Leptospira*.

### *Blocking* L. interrogans *with serum against each recombinant protein decreases bacterial serum resistance*

Based on serum resistance displayed by pathogenic leptospires [51], we prompt to investigate whether blockage of native LIC11711 and LIC12587 in leptospiral cell surface would interfere in bacteria survival upon NHS challenge (Figure 9(b)). When cells were pre-incubated with mouse pre-immune serum and then challenged either with NHS or iNHS, no difference in cell viability was observed; in contrast, antibody-mediated blockage of LIC11711 and LIC12587, separately or in tandem on the bacterial surface, caused reduced leptospiral viability upon challenge (p < 0.05). A more prominent effect upon cell viability was observed when leptospires were treated with anti-LIC12587 antiserum, which is in accordance with the greater interaction of rLIC12587 with complement molecules and regulators. Heating the serum anti-recombinant proteins at 56°C did not affect the ability of the antibody to interact with their respective antigen (data not shown).

## Discussion

The pathogenic species of *Leptospira* enter the host mainly through cuts and abrasions on the skin [1], which expose the ECM. It is known that pathogenic leptospires are capable of binding to structural components of the ECM, such as collagen, laminin, fibronectin and cell-surface receptors, such as cadherins [52,53]. Several leptospiral proteins expressed in *E. coli* as His-tag recombinant proteins have been characterized *in vitro* as putative adhesins that could mediate the binding of the bacteria to the host components [10,54]. The involvement of His-tag on the binding was excluded because many of these recombinant proteins did not interact with the ECM components tested [46,55].

In this work, two novel leptospiral hypothetical lipoproteins, coded by the genes LIC11711 and LIC12587, were selected for validating their expression and function during infection. The *in silico* results, corroborated by immunoblotting with the cell extract of different *Leptospira*, indicate that both proteins are conserved among pathogenic strains. In addition, patients’ serum samples at the onset and convalescent phase of the disease displayed reactivity against the recombinant proteins, suggesting that their native counterparts are expressed and immunogenic during infection.

Recombinant proteins rLIC11711 and rLIC12587 exhibited a dose-dependent binding to laminin, however with a lower affinity in comparison with previously characterized Lsa46 and Lsa77 [9]. Both recombinant proteins were characterized as novel E-cadherin (epithelial) ligands. The involvement of His-tag in mediating these interactions was ruled out since several recombinant proteins do not react with E-cadherin [14]. Recombinant LIC11711 exhibited a saturable dose-dependent binding to E-cadherin with a higher affinity than the previously described Lsa16 [14], and recombinant rLIC12587 also interacted in dose-dependent manner, but without reaching a saturation point. The K_D_ for rLIC11711 and E-cadherin (3.82 ± 0.21 μM) is of the same order of magnitude when compared with rLIC10831 and the same component (K_D_ 2.3 ± 0.3 μM) [56]. Evangelista et al. [57] described the ability of leptospires to disrupt monolayers of EA.hy926 and HMEC-1 endothelial cells *in vitro* by binding into vascular endothelial (VE)-cadherin responsible for cell-cell adhesion in endothelia as a mechanism of host invasion. In this context, binding of rLIC11711 and rLIC12587 to ECM components laminin and E-cadherin *in vitro* may reflect a similar mechanism of leptospires to initially adhere in ECM-exposed epithelial cells.

Target organs provide an immune-privileged site where leptospires may persist for prolonged periods [2]. In this context, a mechanism used by leptospires to reach these sites is the acquisition of host proteases capable of degrading ECM components, complement molecules, and immunoglobulins. It has been previously shown that the pathogenic leptospires are able to capture circulating PLG through multiple surface receptors [14,36,42,58], and subvert the host machinery to convert the zymogen into its active form, PLA, a broad-spectrum serine protease capable of degrading several host components, favoring bacterial dissemination and immune evasion [15,59].

The proteins rLIC11711 and rLIC12587 bound to PLG in a dose and time-dependent manner. The participation of His-tag in these interactions was excluded because we have reported that several recombinant proteins do not bind PLG [42]. Both interactions were demonstrated to occur via kringle domains in PLG molecule and lysine residues in recombinant proteins, as previously described for several leptospiral PLG-binding proteins [14,15,36,58,60,61]. The PLG-bound recombinant proteins could be converted to PLA, whose proteolytic activity was observed by the degradation of the substrate d-Val-Leu-Lys 4-nitroanilide dihydrochloride. However, when human serum was used as unique source of PLG, only proteolytic activity of PLA could be observed for rLIC11711, possibly due to its higher affinity. This data may reflect what happens *in vivo*, in which LIC11711 protein may play a role in PLG acquisition.

It has been described that spirochetes are capable of preventing the lytic activity of host serum and even prevent the membrane attack complex formation as an evasion mechanisms by binding to different complement components and regulators [51,62]. Some leptospiral OMPs displayed *in vitro* binding ability to terminal pathway proteins of complement system or host complement regulators [16,61]. Recombinant rLIC11711 exhibited binding to C8 while rLIC12587 bound to C7, C8, and C9 in a saturable dose-dependent manner. In general, recombinant rLIC12587 showed a lower affinity to C8 and an equal affinity to C9 compared to Lsa23, the first leptospiral protein reported to bind the terminal pathway of complement [16]. Both recombinant proteins, but mainly rLIC12587, were able to capture the complement system components of the serum and favor the survival of *E. coli*, which corroborates with ELISA binding assay of complement from NHS by recombinant proteins. We have reported that NHS plus a His-tagged protein, which does not interact with complement components, has no effect either in causing hemolysis or the formation of C5b9 complex, while Lsa23 that interact with these components is capable of inhibiting hemolysis and C5b9 formation by NHS, showing that the His-tag present in recombinant proteins has no direct influence on NHS [17].

Interestingly, when LIC11711 and LIC12587 proteins were blocked on *L. interrogans* cells by the respective antiserum, a decrease in bacterial serum resistance was observed, indicating the participation of both proteins in leptospiral immune evasion process. These data are similar to the ones we have previously reported, showing that blocking pathogenic *Leptospira* with serum anti-Lsa23, which reacts with complement components, decreased cell viability to complement attack, while anti-Lsa36, which does bind complement components, had no effect on NHS complement attack to *Leptospira* [17]. Moreover, these results show that antibodies against His-tagged proteins have no direct interaction with NHS. The ability of rLIC12587 to bind to complement component C9 may lead to polymerization inhibition, indicating the potential of this protein in inhibiting MAC formation. The rLIC11711 may assist bacterial immune evasion by decreasing opsonophagocytosis through PLA generation, as previously described for *Leptospira* [63].

Vitronectin performs different functions in the human body. This glycoprotein has in its carboxyl-terminal portion a heparin-binding domain composed of approximately 40 mostly basic amino acids [64]. Vitronectin is an important regulator of complement pathway acting as an inhibitor for MAC deposition in cell surface [65].

The competition assay with heparin shows that rLIC11711 interacts with vitronectin, through heparin-binding domain stronger than rLIC12587. It is possible that rLIC11711 also reacts with fibronectin via the same domain. Proteins involved in the process of colonization, invasion or immune evasion may constitute interesting vaccine candidates [66–68]. In addition, it is expected that these proteins will be exposed and conserved in different pathogenic species. Surface-exposed proteins, due to their nature and location, could stimulate a T-dependent immune response and broad-spectrum protection [69].

In conclusion, the proteins coded by the genes LIC11711 and LIC12587, previously genome annotated as hypothetical, are newly described versatile leptospiral proteins, surface-exposed, and expressed during the infection. These proteins have the capability to interact with E-cadherin adhesion molecules, with the fibrinolytic system, and with complement components. Thus, they have the potential to participate at different pathways of leptospiral infection and may be used as diagnostic candidates.
